# Blood-Based Cancer Biomarkers in Liquid Biopsy: A Promising Non-Invasive Alternative to Tissue Biopsy

**DOI:** 10.3390/ijms19102877

**Published:** 2018-09-21

**Authors:** José Marrugo-Ramírez, Mònica Mir, Josep Samitier

**Affiliations:** 1 Nanobioengineering Group, Institute for Bioengineering of Catalonia (IBEC) Barcelona Institute of Science and Technology (BIST), 12 Baldiri Reixac 15-21, 08028 Barcelona, Spain; jmarrura7@alumnes.ub.edu; 2 Centro de Investigación Biomédica en Red en Bioingeniería, Biomateriales y Nanomedicina (CIBER-BBN), Monforte de Lemos 3-5, Pabellón 11, 28029 Madrid, Spain; 3 Department of Electronics and Biomedical Engineering, University of Barcelona, Martí i Franquès 1, 08028 Barcelona, Spain

**Keywords:** liquid biopsy, cancer, biomarkers, non-invasive, circulant tumor DNA (ctDNA), circulant tumor cells (CTC)

## Abstract

Cancer is one of the greatest threats facing our society, being the second leading cause of death globally. Currents strategies for cancer diagnosis consist of the extraction of a solid tissue from the affected area. This sample enables the study of specific biomarkers and the genetic nature of the tumor. However, the tissue extraction is risky and painful for the patient and in some cases is unavailable in inaccessible tumors. Moreover, a solid biopsy is expensive and time consuming and cannot be applied repeatedly. New alternatives that overcome these drawbacks are rising up nowadays, such as liquid biopsy. A liquid biopsy is the analysis of biomarkers in a non-solid biological tissue, mainly blood, which has remarkable advantages over the traditional method; it has no risk, it is non-invasive and painless, it does not require surgery and reduces cost and diagnosis time. The most studied cancer non-invasive biomarkers are circulating tumor cells (CTCs), circulating tumor DNA (ctDNA), and exosomes. These circulating biomarkers play a key role in the understanding of metastasis and tumorigenesis, which could provide a better insight into the evolution of the tumor dynamics during treatment and disease progression. Improvements in isolation technologies, based on a higher grade of purification of CTCs, exosomes, and ctDNA, will provide a better characterization of biomarkers and give rise to a wide range of clinical applications, such as early detection of diseases, and the prediction of treatment responses due to the discovery of personalized tumor-related biomarkers.

## 1. Introduction

The abnormal and uncontrolled cell growth, known as cancer, is considered the second leading cause of death globally. Lately, personalized medicine has been gaining a lot of attention for being one of the most promising areas for cancer therapy. According to the World Health Organization (WHO), the number of deaths owed to cancer in 2015 was 8.8 million globally and in 2014, almost 1.3 million people died from this disease in the EU (World Health Organization, 2018). For the EU, the standardized death rate in 2014 was 261.5 per 100,000 inhabitants, which was lower than the rate for circulatory diseases, but higher than the rate for most other causes of death [1]. The effect of cancer on society is massive, and an early, sensitive, and accurate diagnosis can be considered sine qua non in cancer management, as it can lead to effective therapeutic interventions, reducing the treatment cost and substantially improving patient outcome and overall survival (OS) [2].

Currently, clarification of the molecular landscapes of the tumor is crucial to guide and provide better treatment choices in clinical practice. Tissue biopsies are the current method to access the molecular information of the tumor and are required for the identification of its nature, such as type of cancer, gene and mutation expression, and screening [3]. However, it is fraught with issues, such as a required invasive surgical extraction, which could cause discomfort, pain, and risk for the patient. There are several clinical risks that are inherent in procedures and the possibility of surgical complications. Moreover, some tumors are difficult to access in some anatomical locations, which are not accessible for a biopsy; and, in some cases, the extraction of it may augment the risk of metastatic lesions [4]. Sometimes the amount of tissue extracted in not enough for all the required tests and it needs to be repeated, which is also needed if the tumor is not homogeneous and/or is evolving along with the disease. Moreover, the solid biopsy methodology involves a high financial cost, it is time consuming, and requires an operating theater. So, even if different metastatic locations could be biopsied simultaneously, there could be a delay in the start of the treatment due to the analysis of the samples and it might compromise the prognosis [5].

Moreover, the evolution of the tumor needs to be monitored at separate times of the disease for an efficient treatment of the illness, so solid biopsies cannot be considered again for being highly invasive, and, normally, optical methods are used; however, they do not provide complete information about the tumor. Radiology is also widely used; however, excessive levels of radiation could generate a health risk for the patient. Non-radiation approaches, such as magnetic resonance imaging (MRI) scans, are considered inconclusive and inefficient for the minimal residual disease (MRD) detection (reviewed in detail by [6,7]), and, also, due to the limited information provided. Moreover, safety can be questionable, for instance, regarding sampling of tumors surrounding major vessels or in eloquent regions of the brain, or in patients with major comorbidities [8]. Therefore, these types of techniques are clinically unfeasible and unable to encompass the temporal and spatial heterogeneity of the tumor, i.e., several populations of cancerous cells (with different genetic variations) might exist in different regions among the tumor spatial heterogeneity or have considerable differences between the original lesion and a recurrence, that could be either a local or distant temporal heterogeneity [2,9,10].

Considering the temporal and spatial heterogeneity of the tumor, the most logical step to follow is the extraction of several biopsies from the patient’s initial lesion and their respective metastasis; however, all the drawbacks discussed previously make this possibility unfeasible. Consequently, there is an urgent need to search for minimally invasive biomarkers in order to detect and monitor the disease progression at several time-intervals throughout the treatment. One promising alternative to tissue biopsy is the study of the communication between cancer cells, or any other cancer-related biomolecule (cells, nucleic acids, proteins, microvesicles, etc.) and their microenvironment.

By understanding this approach, researchers and clinicians could be able to foresee the behavior and response of tumor cells to patient-specific therapies, allowing an improvement of outcomes and OS rate. Liquid biopsy, defined as the capture of tumor-related biomarkers in a fluid sample, has been extensively studied and is growing in popularity due to its minimal invasiveness, low consumption of reagent, and ease-of-use.

During the apoptosis and necrosis of tumor cells, these biomarkers are released into the bloodstream, facilitating and promoting metastatic activity in nearby and/or distant organs. Lately, this approach has been considered for applications in the early diagnosis of tumors, therapeutic guidance, and recurrence monitoring. These advantages are possible due to the abundant information that it can provide [11].

**Figure 1:**
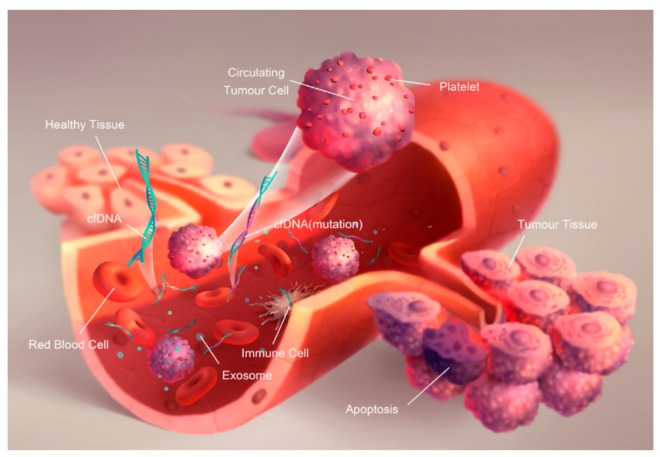
Schematic of the origin of cell-free DNA (cfDNA), circulating tumor cells (CTCs), and exosomes in the blood by [12], licensed under CC BY-NC-ND. The final, published version of this article is available at http://www.karger.com/?doi:10.1159/000458736.

According to several research studies, the liquid biopsy approach has been focused on the analysis of circulating tumor cells (CTCs), circulating tumor nucleic acids (ctNAs) and/or tumor-derived extracellular vesicles (exosomes), which have been shed from tumors, and their metastatic sites, into the bloodstream, saliva, urine, cerebrospinal fluid (CSF), among other peripheral fluids of cancer patients. Figure 1 shows the scheme of the different tumor-related biomarkers aforementioned and their presence in blood—further reviewed in ref. [12]. The concentration of these biomarkers in the bloodstream might contribute to an earlier detection of the stage of cancer and a more favorable prediction of the prognosis in patients. 

It has been found that levels of these biomarkers increase in patients with several malignant types of tumors, such as prostate, colorectal, stomach, lung, breast, among others. Most studies have been done in patients with late-stage cancer, usually stage III and IV, mainly due to the considerably higher volumes of the aforementioned biomarkers in their blood. 

In patients with prostatic cancer, a study showed that more than 5 CTCs per 7.5 mL of blood [13], and an approximate circulating tumor DNA (ctDNA) concentration of 437 ng/mL of blood vs. healthy patients (99 ng/mL of blood) [14] could be considered unfavorable, so it could reduce considerably their OS. Regarding colorectal cancer, a study revealed a significantly broad range for ctDNA in patients suffering from it (22–3922 ng/mL of blood), in comparison with healthy ones (5–16 ng/mL of blood) [15]. Several gastrointestinal diseases could also lead to an increase in ctDNA, even if considered benign or malignant. Normal controls presented ctDNA levels of 14 ± 3 ng/mL, which are remarkably lower than those obtained for malignant and benign diseases, 412 ± 63 ng/mL and 118 ± 14 ng/mL, respectively (Shapiro et al., 1983). Lung cancer patients and control patients, unlike other types, demonstrate significant differences in ctDNA levels since stage I, i.e., 318 ng/mL and 18 ng/mL respectively [16]. Among other studied types of cancer, women suffering from metastatic breast cancer presented two or more CTCs per 7.5 mL of blood, in comparison to healthy women (0.1 per 7.5 mL of blood) [17].

Even though CTCs and ctDNA can be considered as an attractive tool for early detection and diagnosis of cancer, recent studies have shown that the sensitivity and specificity of tests could be considerably improved if coupled with conventional biomarkers for several types of tumors. For instance, a test combining ctDNA with carcinoembryonic antigen (CEA) for colorectal cancer, and pancreatic carcinoma, ctDNA with prostate-specific antigen (PSA) for prostate cancer [14], and ctDNA with microsatellite alterations for lung cancer [16], could be a potentially useful tool for a better understanding of the disease progression and early-stage diagnosis.

In this comprehensive review, the source, characteristics, and technology of detection of CTCs, ctNAs, and exosomes, as well as their use in the diagnosis, recurrence monitoring, and prognostic assessment for tumors is described.

## 2. Concept of Liquid Biopsy

In order to properly discuss the liquid biopsy approach in detail, it is pivotal to understand the different types of cancer-related biomarkers and their respective biogenesis. For several years, CTCs, extracellular vesicles called exosomes, and ctNAs, such as ctDNA and microRNAs (miRNAs), have been considered the main biomarkers in the liquid biopsy approach for cancer [18]. As mentioned before, these biomarkers are shed off into peripheral fluids from the tumor site, so they can be detected and analyzed in order to improve the clinical settings of a patient tumor.

Indeed, the rapid turnover of cancer cells is assumed to be the cause of the constant release of tumor-derived nucleic acids, vesicles, and viable CTCs into the circulation. Thus, the ability to detect and characterize circulating cell-free tumor DNA (ccftDNA), tumor-derived RNA (predominantly microRNAs (miRNAs)), and CTCs, has enabled doctors and surgeons to analyze the evolution of the tumor several times and, most importantly, in a non-invasive approach.

### 2.1. Circulant Tumor Cells (CTCs)

#### 2.1.1. Biogenesis

While examining peripheral blood under a microscope, Thomas Ashworth discovered CTCs in 1860. Therefore, a theory based on the penetration of tumor cells into the vessel wall and their respective entry in the bloodstream was then proposed [19]. Particularly, most CTCs were found to be accidental circulant cells, i.e., being passively or actively pushed by external forces, including tumor growth, and mechanical stress during surgical operation [20]. CTCs are, indeed, tumor cells that are mostly shed from primary lesions during their formation and early growth. They circulate through the bloodstream to potential metastatic sites, either as a single cell or in clusters, becoming the main mechanism for metastasis [21,22].

In order to capture and analyze CTCs, it is important to understand their major obstacles: Their complex surface and overall heterogeneity make them extremely rare. One of them is that they are, quite literally, one in a million or billion (approx. 1 cell per 10^9^ blood cells in patients with metastatic cancer), among other cells in the blood [8]. Another challenge has to do with the variety of their surface protein expressions, sizes, and physical characteristics, depending on the type and stage of cancer. Additionally, the number of captured and detected cells in the blood of patients with any type of cancer has been correlated with treatment outcomes and OS. The capture of these biomarkers could be done in several ways, including immuno-recognition, separation based on size or stiffness, among other chemical or physical recognition methods.

#### 2.1.2. Technologies and Strategies for Detection

**Figure 2:**
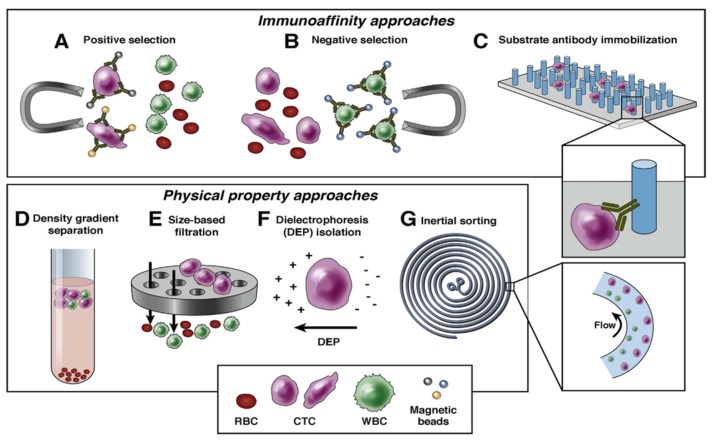
Biological and physical approaches of enrichment. Retrieved with permission from [23]. Copyright 2016, Elsevier.

The main challenge in the detection of CTCs is the identification and characterization of single tumor cells, which are remarkably hard to locate, compared to the millions of other hematopoietic cells. As mentioned before, its rarity makes it extremely difficult, along with its low abundance and concentration, and even more in early-stage cancers, as it is at even lower concentrations. Therefore, it requires extremely sensitive and specific analysis methods, which have been developed in order to achieve maximum CTC collection. The enrichment and detection methods of CTCs can be classified based on their biological and physical properties, as Figure 2 describes below.

##### Biological Properties

Detecting CTCs based on their biological properties is mostly related with proteins present in the cell membrane. To target these molecules immunoselection-based procedures are used, with two different approaches: Antibodies against tumor-associated antigens, which is called positive selection, or against the common leukocyte antigen, which is called negative selection. A very recognized technique is the immunomagnetic isolation, which consists of the attachment of an antibody to a magnetic bead in order to target an antigen. The formed antigen–antibody complex can then be isolated by exposing the sample to a magnetic field. Based on several studies, antibodies against the epithelial cell adhesion molecule (EpCAM) are commonly used for positive selection.

*CellSearch* is the only EpCAM-based assay that is FDA-approved, and it is also considered the “gold standard” for CTC detection. As mentioned, the main goal of this technique is to enumerate epithelial CTCs biomarkers, such as CD45^−^, EpCAM^+^, CK 8^+^, and 19^+^, among others, in whole blood [24]. The detection of CTCs is closely associated with a diminishment of the OS in patients that are being treated for metastatic breast, prostate or colorectal cancer. As this technique would not give a “complete” landscape of the tumor dynamics and patient monitoring, it should be performed and analyzed jointly with other clinical information derived from other types of diagnostic tests, such as ctDNA genetic tumor study, imaging, and the patient’s medical history, in order to give a proper management of the prognosis of the cancer.

Hayes and colleagues have utilized *CellSearch* for the detection of metastatic breast cancer and reported that a baseline of 5 CTCs in 7.5 mL of blood would be associated with poor OS among the 177 patients evaluated in the study, further reviewed in [25]. However, there are several cases in which cancer patients would lack EpCAM expression; hence, a different antibody, or even isolation method, would be necessary in order to capture CTCs in circulation [26].

There have been multiple developed technologies using several types of capture devices. Among them, there are microfluidic-based platforms, which would allow a better control of small volumes of reagents during several processes. In 2007, Nagrath et al. [27] reported that CTCs, from whole blood, could be detected and captured using a microfluidic device based on an anti-EPCAM approach. Compared to previously reported studies, this approach isolated CTCs with better levels of enrichment and sensitivity. Currently, several microfluidic platforms have used a combination of immunoaffinity- and size-based approaches in order to develop an integrated system capable of improving the purity and recovery levels of CTCs. For instance, Ozkumur et al. [28] described a microfluidic-based chip for CTC separation, known as *iChip*, which combines magnetic separation, hydrodynamic sorting, and inertial focusing of CTCs from blood. 

##### Physical Properties

The key advantage of physical properties is that they allow a label-free separation of CTCs. These biomarkers have shown particular physical properties, including heterogeneity, buoyant densities and larger size (7–30 µm). Despite their evident heterogeneity, several CTC isolation platforms have been proposed for their capture from blood with considerably higher purity and recovery rates. Some of the main CTC-enrichment methods that are based on the physical properties include inertial sorting, density gradient separation, dielectrophoresis isolation, size-based filtration, and photoacoustic flow cytometer, among others [23]. 

##### Density Gradient Separation

In most studies, blood samples are diluted, put as a “coat” over the media and finally centrifuged in order to allow density-based separation. Some of these methods include *Ficoll-Hypaque* media, as well as the *OncoQuick* setup from Pharmacia-Fine Chemicals and Greiner BioOne, respectively. The last is supposedly better due to an incorporation of a porous membrane to decrease the number of contaminating blood cells with similar densities to CTCs. 

##### Size-Based Filtration

This type of platform is used for the enrichment of CTCs based on their slightly larger size, compared to other hematopoietic cells. For instance, size-based isolation of epithelial tumor cells was employed to enrich CTCs from blood samples employing a polycarbonate-based membrane filter with cylindrical pores of 8 µm, approx. [29]. Nevertheless, cell fixation potentially limits subsequent genomic analyses. An alternative to this platform is the microfluidic approach involving microtraps, which leverage the size and deformability of CTCs, such as CytoQuest™ from Abnova Diagnostics (Taipei, Taiwan). Using size-based filtration methods also has several drawbacks, including relatively low flow rates and non-specific capture of cell debris.

##### Inertial Sorting

This approach employs hydrodynamic forces, which focus on blood cells at distinct streamlines in fluid. Sollier et al. has developed a size-based separation method using a combination of inertial focusing and microfluidic vortices. The basis of this approach consists of the use of inertia and fluid flow effects in order to separate different particles within a channel [30]. However, some major drawbacks are the possibility to isolate different blood cells (not the target) of similar sizes and a loss in detection if the CTC size is not within the working size range. 

Altogether, with the recent advances in engineering and technology, several next-generation microfluidics platforms have been produced in order to recover CTCs with higher levels of purity and the potential to be cost-effective in the clinical setting.

#### 2.1.3. Diagnosis and Recurrence Monitoring for Therapies

At present, the clinical value of CTC analysis remains controversial, even though there is proofing evidence indicating both a prognostic value and a correlation between the abundance of tumor cells in the blood of cancer patients and the response to therapy and, thus, treatment outcomes and OS.

##### Prostate Cancer

Prostate cancer presents particular complications in both patient monitoring and drug development. This peculiarity is based on two important aspects: (1) The fact that standard imaging approaches employed for the evaluation of the disease in bone, which is the most common location for cancer spread, have not been standardized yet; (2) The prostate-specific antigen (PSA) levels, which is the most common biomarker in the disease, might not reflect an accurate status of the disease.

An important finding for prostate cancer was that the second aspect aforementioned was verified. According to several studies, the correlation between the number of isolated cells and the overall disease status (based on PSA levels and disease spreading in bone) was discovered to be low. This reflected that the number of isolated cells represent an intrinsic property of the tumor. Based on several studies [31,32], patients presenting less than 5 CTCs in 7.5 mL of blood can be considered favorable and have a better prognosis than patients with more than 5 CTCs. Based on this, patient-specific treatments can be carried out in order to monitor and diminish the abundance of tumor cells for a better outcome.

##### Breast Cancer

Based on the WHO and several cancer-related studies, breast cancer is the most widespread neoplasm, with 5.2 million cases worldwide. Hence, there is an urgent need for improvements in the disease diagnosis and its patient-specific management. The majority of the information established on the CTC’s clinical setting is derived from several studies using a technology called *CellSearch*, which has demonstrated its importance in the prognosis of patients with numerous types of solid tumors, including prostate, colon, and breast cancer. Based on those results, a CTC cut-off level of ≥5 cells per 7.5 mL of blood was identified and correlated with a higher risk for disease progression and a considerably low OS [17].

##### Pancreatic Cancer

Based on their phenotype, the great majority of solid neoplasms formed in the pancreas are pancreatic ductal adenocarcinomas (PDACs). PDAC is diagnosed in most patients at an advanced stage, making it extremely difficult to foresee a good prognosis. Due to the fact that these tumors are mostly aggressive, and, according to several studies, they have a considerably poor response to cytotoxicity-based and targeted therapies, only 7% of patients survive for approx. 5 years [23]. One of the main challenges of PDACs is that the symptoms appear after the cancer has reached its threshold of manifesting by itself; therefore, is quite complicated to detect at an early stage. Several studies have suggested that the use of different technologies, such as *CellSearch* and CTC-Chip, based on the detection of CTCs in blood can contribute to an early detection. Allard and colleagues carried out a study using *CellSearch* in healthy subjects, and patients with a variety of metastatic carcinomas, including PDAC. Based on the CTCs counts, PDAC was the most complicated carcinomas to detect, presenting the lowest numbers of CTCs (mean, 2 ± 6 cells in 7.5 mL of whole blood) compared with prostate, ovarian, breast, and lung, among others [33]. However, they concluded that CTCs are extremely rare in healthy subjects but present in various metastatic carcinomas, so the monitoring of CTCs in blood before any symptoms may help in the early diagnosis of these diseases.

### 2.2. Circulant Tumor Nucleic Acids (ctNAs)

#### 2.2.1. Biogenesis

ctNAs encompasses the presence of circulating tumor DNA (ctDNA) and circulating tumor RNA (ctRNA) fragments from tumor cells in the bloodstream. Before discussing ctNAs in full detail, it is necessary to understand their biogenesis. 

Cell-free DNA (cfDNA) is defined by [34] as the portions of extracellular occurring DNA (eoDNA) that is not related to any subcellular or molecular structure. This particular biomarker has increasingly gained attention due to its presence in body fluids, and for being a part of several pathological and physiological processes, including cancer, coagulation, etc. In cancer patients, a small fraction of cfDNA originating from cancer cells is referred as ctDNA [35], and it might carry the same genetic alterations and mutations as those existing in the primary tumor [2]. Therefore, ctDNA could potentially provide an opportunity for a non-invasive evaluation and prognosis of cancer. 

In normal physiological conditions, necrotic and apoptotic cell debris are removed from the tissue by infiltrated phagocytes. This process, combined with the accelerated cellular turnover, is not as effective in cancer cells, which will lead to an exponential increase of necrotic and apoptotic cell debris. In consequence, the release of the biological content within apoptotic and necrotic cells into the bloodstream, including ctDNA, is considerably higher than in normal conditions.

As mentioned before, the CTC-based methods have several advantages, such as having high specificities, and low signal-to-noise ratios, especially in the early detection of cancer. In comparison with CTC detection, the ctDNA-based assay can provide a better detection of a patient-specific disease and treatment. Another advantage of ctDNA is that it can provide a real-time snapshot of the patient’s disease status, which is not possible in the traditional “tissue biopsy” approach. Additionally, as observed in CTCs, ctDNA can also display a higher sensitivity for early detection of cancers. In contrast to CTC capture, the ctDNA enrichment process does not depend on using specific equipment [36].

The other group within the ctNAs is the ctRNA, which comprises two types of noncoding RNAs (ncRNAs): Long noncoding RNAs (lncRNAs)—longer than 200 bp, and microRNAs (miRNAs)—shorter than 200 bp [34,35]. lncRNAs are present in the nucleus, where they interact with other NA and proteins, and play a key role in the regulation of gene expression [37]. lncRNAs are also present in several processes, such as the splicing of regulatory proteins in the cytoplasm, in order to greatly influence the splicing of messaging RNA, and also the stability of mRNA, based on the translation factors [38]. One of the remarkable aspects of lncRNAs is that they could isolate miRNAs so they could not bind to their respective targets [39,40]. Unfortunately, very little information is given about lncRNAs and only a fraction of them have been experimentally analyzed. Tumor-related lncRNAs could be used as a diagnostic biomarker and is considered one of the “newly” investigated biomarkers to provide several therapeutic approaches [41]. 

Another well-known ctNA biomarker in the liquid biopsy approach is the microRNA (miRNA), which is a small (18–25 nucleotides), endogenous, single-stranded RNA [42]. miRNA modulates the expression of nearly 30% of protein-encoding genes in humans. If there is a modification in the expression or a dysfunction, a number of biological processes can be affected, which can trigger a tumorigenesis and/or affect the disease progression [43].

The miRNAs can also be spread by cells into the bloodstream in a stable form, and some of them can distinguish cancer patients from healthy individuals [44]. According to several studies, the miRNA biogenesis has been lengthily described over the past years; however, the mechanism of incorporation into multivesicular bodies, such as exosomes, is not well known. One of these possible mechanisms, followed by its secretion in blood, was described in [45], in which they focused on a strategy, known as Dicer–knockout, in order to explain this secretion process. Although the correlation of both miRNA processing and exosome incorporation during the exosome biogenesis is relevant, it is one of the issues that this approach has. Besides the exosome-encapsulated miRNA, there are other types of secreted non-exosomal miRNA that are also present in circulation, such as those secreted passively from cells and others stabilized by binding to RNA-binding proteins and high-density lipoprotein complexes [46]. The evidence of tumor-associated RNA in cell-free plasma—or serum—not only provides new targets for cancer monitoring and detection, but also opens up the opportunity of non-invasive gene expression profiling for cancers [47,48]. 

Based on several studies, and the information stated above, it is valid to state that circulating ncRNAs have a pivotal role in different biological processes, including tumor biogenesis, progression, and metastasis. Tumorigenesis is usually related to both an over-expression of several oncogenes and the down-regulation of tumor-suppressor genes [45]. It is known that tumorigenesis is frequently associated with an over-expression of oncogenes, as well as a down-regulation of tumor-suppressor genes. miRNAs in liquid biopsy also have several advantages over other types of biomarkers, including tissue-specific expression and ease of detection. Therefore, both lncRNAs and miRNAs might be considered as potential biomarkers and “game-changers” for diagnosis, prognosis, and patient-specific therapy [49]. 

#### 2.2.2. Technologies and Strategies for ctNAs Detection 

**Figure 3:**
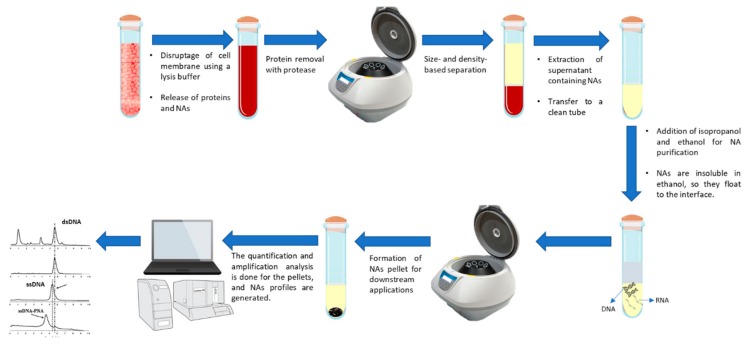
Commonly used extraction procedure for nucleic acids. Prior to circulating tumor nucleic acids (ctNAs) detection, several methods have been utilized in order to properly isolate these biomarkers.

Before covering the section of ctNA detection, it is pivotal to discuss the extraction and purification of NA, which follows four main stages: Cell lysis, protein denaturation, extraction of proteins from NA, and precipitation and dissolution of NA, more detailed information is provided by [50]. Figure 3 shows the step-by-step procedure for DNA and RNA extraction. 

Table 1 summarizes some of them, and they are further reviewed in detail in references [51,52,53,54].

Several procedures are available for quantification of nucleic acids. The main division for the quantification of NAs is based on the type of measurement, i.e., it could be a spectrophotometric quantification, fluorescent dye-based reading, or real-time amplification [55]. The first one is based on the use of a spectrophotometer, which is the most common device to quantify NAs, and is determined through the reading of nucleosides bases absorbance. The second approach is based on a fluorescence emitted by a dye intercalated inside of DNA and RNA helix structure [55]. Real-time PCR could be an alternative approach for the estimation of NA concentration based on targeting specific sequences [56]. Since the data obtained with different methods are not directly comparable [57], the same approach must be chosen to properly compare data obtained from different reports. 

Although cancer patients present higher cfDNA levels than healthy ones, the concentrations of overall cfDNA vary considerably in plasma or serum samples. As mentioned before, ctDNAs is a small variable fraction of cfDNA, usually from 0.01% to slightly more than 50% [6]. Some studies have hypothesized about the presence of ctDNAs in blood are mainly due to a release of the content of apoptotic or necrotic tumor cells or from tumor-derived multivesicular bodies, such as exosomes [58]. In the blood of healthy patients, the length of cfDNAs ranges between 70 and 200 bp, having also a concentration range between 0–100 ng per ml of whole blood. However, ctDNAs from cancer patients have lengths ranging from 200 bp to more than 1 kb, with concentration ranges from 7 to 18 ng per mL of blood [59]. Their half-lives are usually from 15 min to a few hours, being removed by the liver and kidney [60].

For an early detection of cancer, only PSA is extensively used as a blood test [61]. There are no commercially available blood tests for cancer detection and the ones that are, have the tendency of being highly invasive—such as colonoscopy and cytology. Liquid biopsies could greatly influence the prognosis of patients due to a higher diagnostic specificity and sensibility [62,63]. Nevertheless, a very important issue is based on the nature of the tumor, as mentioned in previous sections. The same gene variations could be present in several types of tumors, so the test has to be done jointly with other diagnostic tools, in order to make a proper diagnosis and prognosis. Cohen and colleagues proposed a blood test, known as *CancerSEEK*^®^, that could improve several issues regarding liquid biopsies. This test is based on combined assays for the detection of genetic variations and 8 protein biomarkers, but it also has the capacity of both identifying and locating an early-stage tumor with its respective organ of origin. According to the study, *CancerSEEK* was applied to 1005 patients with non-metastatic cancers—e.g., liver, stomach, lung, among others in which 70% of the median were positive and the range of sensitivities for five cancer types (liver, ovary, pancreas, esophagus, and stomach) were significantly high (70–98%), considering that there are no screening tests available. This study is further reviewed in reference [64]. 

Based on the small size and low concentration of ctDNAs in blood, highly sensitive and target-specific techniques have been developed in order to detect them. In principle, detection strategies can be classified into two groups: Targeted approaches, which aim to detect specific mutations in a pool of predefined genes, or Untargeted approaches, which have the goal of screening the genome and discovering new specific aberrations, such as conferring resistance to a specific targeted therapy [65]. Recently, there has been a need for ultrasensitive technologies capable of detecting the smallest amounts of ctDNA in the large abundance of normal cfDNA, which are needed for early detection of cancer or minimal residual disease (MRD). 

##### Targeted Approaches (Mutations of ctDNA)

Physiological aspects, including tumor burden and biological mechanisms, have a crucial effect on the quantity of detectable ctDNAs [66]. Accordingly, to evaluate the clinical benefit of ctDNA, it is important to understand the technical aspects of its detection. Due to recent technological advances, several methods, including single nucleotide polymorphism (SNP) analysis, genome-wide association studies (GWAS), and next-generation sequencing (NGS), are now available for the detection of genetic mutations in different cancer. These methods have been used to deal with some difficulties that were evident in the accuracy and sensitivity of tumor genomes detection, as well as providing a better molecular landscape of the tumor biology [67]. The SNP array is based on the use of unique nucleotide sequences as probes, which would hybridize with different fragments of single-stranded DNAs. Furthermore, GWAS employs a chip-based microarray technology in order to facilitate the analyses of more than one million SNPs, opening up the possibility of detecting tumor-specific DNAs, allowing the development of blood-based diagnostic tests for cancers [68,69]. 

##### Untargeted Approaches (ctDNA Methylation)

Untargeted methods based on the study of DNA methylation can be classified as site-specific detection and genome-wide methylation detection [70]. Following several processes, including bisulfite conversion and enrichment ctDNA methylations, the detection can be facilitated employing different PCR amplification methods, including the conventional methylation-specific PCR (MSP) [71], quantitative multiplexed methylation-specific PCR (QM-PCR) [72], and a modified version of PCR known as methylation on beads (MOB) [73].

Conventional MSP has been used several times in the detection of methylated ctDNA [71]. According to Wong and colleagues, only 5 mL of peripheral blood is needed to carry out this method and can also be used in clinical applications as non-invasive measurements [74]. The main working principle of MOB, which is an improved version of PCR, is the integration of three different processes in a single tube: Extraction of DNA, bisulfite conversion, and conventional PCR. This is carried out using DNA carriers, consisting of superparamagnetic iron oxide nanoparticles within silica microparticles [73].

#### 2.2.3. Diagnosis and Recurrence Monitoring for Therapies

In recent decades, several studies have focused on the use of ctDNA methylations as key biomarkers for an early detection of tumors, their respective screening, and for monitoring patient’s response to therapies against cancer progression. As mentioned before, the main issue is the identification and capture of low amounts of ctDNA in blood, considering the vast amount of cfDNA existing in this media.

Specific genetic variations in tumor cells can provide a better understanding of the physical conditions of patients, as well as their specific treatment responses. By detecting tumor-specific alterations in DNAs existing in the peripheral blood, the dynamic changes in tumor cells could be identified. One remarkable distinction for liquid biopsy of NA is that the workflow is significantly different from CTC or exosome analysis. Circulant NAs have the great advantage that their capture and detection can provide a meaningful analysis, i.e., just by verifying their presence and quantity, there is a remarkable contribution to clinical applications and the development of patient-specific therapies [11].

##### Monitoring of MRD

Currently, the prediction of which patient is or is not “disease-free” after surgery strongly depends on clinical and pathological factors. If not detected early, untreated MRD could lead to cancer recurrence. This would consequently lead to high-risk patients being treated with adjuvant chemotherapy, which might be unnecessary because the patient could have been cured with only surgery or radiotherapy [75].

The detection of cfDNA after surgery or radiotherapy would be an indicator of MRD; therefore, the liquid biopsy approach could be used to identify patients who would or would not benefit from adjuvant therapy. The detection of cfDNA after finishing a surgery or radiotherapy treatment, would indicate the presence of micrometastasis and a very high risk of relapse; thus, a neoadjuvant therapy might be necessary. This information could be used to perform patient-centered molecular diagnostics [7].

##### Monitoring Resistance Evolution to Drugs

It must be taken into account that tissue biopsy reveals only a small fraction of the tumor heterogeneity, especially in patients with metastatic cancer. It is fair to say that there has been no investigation of an effective method capable of successfully evaluate the molecular evolution of the disease, throughout the therapy, in patients with multiple metastatic lesions. In contrast, molecular changes that are related to drug resistance can be identified in an early stage by using ctDNA analysis, and it can be easily performed several times to the same patient at different time intervals [75].

### 2.3. Exosomes

#### 2.3.1. Biogenesis

The transport of biological materials across different membranes is a critical process in order to maintain cell homeostasis. It consists of an active and passive exportation through microparticles, such as exosomes, that maintain a proper assortment of relevant micro- and macromolecules.

When culturing sheep reticulocytes at McGill University, Pan and Johnstone discovered exosomes, for the first time, and reported them in 1983 [76]. These biomarkers can be defined as small, membrane-bound vesicles that are shed from cells for exchanging molecular information between cells, and can act as transport vehicles for a number of biomolecules, such as NA and proteins [18,77] suggesting that they are able to modify the activity of recipient cells. They tend to be in the tens to hundreds of nanometers in size (30–100 nm) and due to the protection of their content from degradation by a lipid bilayer, they have a remarkable advantage over other biomolecules [78]. Additionally, their concentration in blood is usually above 10^9^ individual exosomes per mL of blood and the total amount depends on the tumor burden and stage [18,79].

As previously mentioned, traditional biopsies rely on accessing the tumor cells; however, if exosome-based approaches are used, the respective studies can be applied to subcellular particles and their respective cargos, giving a better elucidation of the tumor landscape. Exosomes, in comparison with CTCs and ctDNA, have several advantages in several aspects, such as their homogeneous size distribution [80], and due to their particular form, they can be distinguishable by using electron microscopy [81]. Moreover, by expressing specific surface proteins, exosomes can present the initial cell markers and their respective target cells [82], which can be employed as a diagnostic tool for many diseases, based on their presence in several body fluids, and their stability in the circulation [83]. Moreover, tumor-specific RNA contained in exosomes can represent their parent, as well as their protein profile and architecture [83]. Finally, exosomes are quite inert, but they can fuse with the cell membrane and either deliver a drug and/or change the recipient cell biology. This feature makes them potentially applicable as nanoscale drug delivery vehicles or gene therapy vectors. 

**Figure 4:**
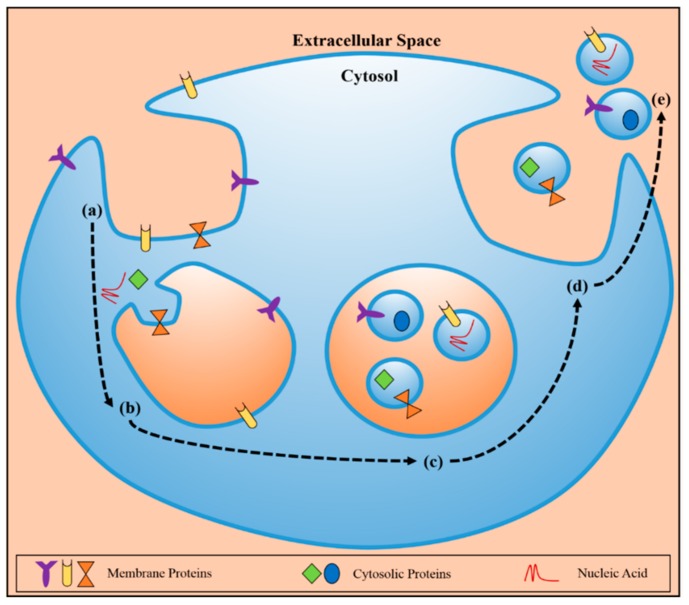
Schematic representation of the formation of exosomes and their respective release to the bloodstream by [78], licensed under CC BY 4.0.

As it can be seen in Figure 4, the endocytosis process starts (a). and leads to the creation of early endosomes with a molecular cargo based on membrane proteins and NAs (b). This new formation will end up forming several particles, known as MVBs, which will contain the cargo-encapsulated exosome (c). After the fusion with the plasma membrane (d). the exosomes are secreted to the extracellular space (e). maintaining the original characteristics and topology to protect NAs and proteins, so they can arrive at several locations, either nearby or distant, and exchange molecular information with other biological structures [78].

#### 2.3.2. Technologies and Strategies for Detection

Due to the fact that there is no “gold standard” available for the isolation and purification of extracellular vesicles (EVs), it is not valid to state an optimal method that could be used uniformly. To carry out a proper planning, design considerations for biological normative, and maximization of the significance of EVs, a series of protocols for providing data and attributing functions to EVs was proposed by the International Society for Extracellular Vesicles (ISEV), which is a network of expert scientists in the field of EV biology. For a more detailed information about the required criteria and the minimal characterization of EVs, based on the technology used nowadays, refer to [84,85].

The segregation of EVs, such as exosomes, employing methods, such as ultracentrifugation, is usually combined with sucrose density gradients or sucrose cushion in order to float the relatively low-density exosomes, which could give a higher enrichment improvement of exosomes. As mentioned before, ultracentrifugation is considered the most efficient approach for the isolation of EVs. It has several advantages over other techniques, including its low cost and convenience; most laboratory settings have them, and its spinning capacity of a wide range of volumes, up to 100 mL [86]. Nevertheless, it presents a series of issues that affect the effectiveness of the technique. One of its major drawbacks is the co-purification of protein aggregates and lipoproteins [87], which are not associated with EVs; though it might be interpreted as an enriched EV for the subsequent downstream application and analysis. The approach of using ultracentrifugation in combination with density gradient mechanisms is based on the need to overcome the co-purification problem presented above. This is due to a subsequent purification of EVs using a sucrose density gradient for a better separation [88]. However, the main disadvantage of this approach is that similar floating densities among structures are indistinguishable. Therefore, this strategy is generally unmanageable and might be convoluted by different biomolecular structures with comparative sizes and floatation densities, such as HIV virus particles [89].

An isolation kit, known as ExomiR, created by Bioo Scientific, could basically remove all cells, platelets, cell debris with only one microfilter and entrap all vesicles that are larger than 30 nm on the following microfilter, in which there is a pressure in order to push the fluid [90].

It is claimed that ExoQuick, a reagent released by System Biosciences, can be added to serum or urine in order to precipitate the exosomes of a certain size (usually between 60–150 nm). The isolation process is very fast, but it lacked specificity toward exosomes; therefore, it can re-mix with non-exosomal content with comparable size [91]. 

Another approach for the improvement of EVs enrichment is the immunoisolation, in which the EVs are subjected to functionalized antibody-coated latex [92] or magnetic beads [93], permitting a biomarker-based separation. One of the main advantages of this method is its specificity to a selected antibody, which will target only EVs with a particular surface marker and avoid unspecific binding to cell debris or other biological structures. In comparison to currently used methods such as ultracentrifugation, density gradient, and ultrafiltration. Immunoisolation technique presents higher yields in the isolation of colon cancer-associated EVs [94]. Nevertheless, it presents the same limitations as in the detection of CTCs and ctNAs. The targeted surface markers could also be present in several subpopulations of EVs. Therefore, it should be used in combination with other enrichment and detection techniques in order to be completely sure about the EVs biogenesis and the organ of origin.

Many exosome-based enrichment methods have been developed on-chip in order to counteract the poor yields and lower the process times obtained in current methods, such as ultracentrifugation. Some of the physical filtration methods used for their capture are ciliated micropillars [95], immunomagnetic isolation [96], immunoisolation on beads [97], and immuno-affinity with nanoshearing [98].

Regarding the on-chip analysis of exosome-related biomarkers, there are many advantages when compared to standard protocols, including high throughput, sensitivity, and automation. Some analytical methods are based on surface proteins analysis by colorimetric detection [98], immunoelectrophoresis [99], qPCR [100], on-chip ELISA detection [101], and mass quantitation [102].

The precise measurement of exosome purity and quantity has been considered one of the principal challenges in its biology. One way to measure the purity, based on several studies, is to quantify their specific markers, either antigen or protein, as a ratio of its concentration using immunoassay-based approaches, such as ELISA [103]. Optical, as well as non-optical methods such as dynamic light scattering (DLS), and surface plasmon resonance (SPR), respectively are being currently employed for quantifying exosomes [104].

#### 2.3.3. Diagnosis and Recurrence Monitoring for Therapies

In contrast with the use of CTCs and ctNAs, the use of the tumor-related exosome as a prognostic biomarker and for treatment guidance mainly depends on its protein and miRNA expression profiles. Some exosome-specific biomarkers include proteins associated with the biogenesis of endosomes and exosomes, EpCAM, and heat shock proteins [80]. The reason behind using these proteins as exosome-based biomarkers is their use in exosome isolation, based on immunoaffinity approaches, and to measure their purity by using several analytical techniques, such as Western blotting analysis [105], transmission electron microscopy (TEM) [106,107], atomic force microscopy (AFM) [108,109]. Based on exosome detection and isolation techniques mentioned above, the subsequent proteomic-based analyses of tumor-derived exosomes led to the identification of potential exosome-related biomarkers that can be used in several types of cancer, including breast, prostate, pancreatic, and glioblastoma [110].

EVs that are separated from plasma contains specific proteins corresponding to several types of cancer, among them, prostate cancer. Biomarkers, such as tensin homolog and survivin, have been identified in prostate cancer patients and the levels of both are considerably higher than in healthy subjects [111,112].

Based on several studies in urological cancers, the ncRNA contained in EVs have gained a lot of attention. The increased stability or specific packaging of ncRNA into EVs is the main cause of the high levels existent in plasma of cancer patients. Bryant and colleagues [113] have described the differential expressions of several EVs-related miRNA in serum and plasma (Further reviewed in [113]).

Based on several studies in urological cancers, the ncRNA contained in EVs have gained a lot of attention. The increased stability or specific packaging of ncRNA into EVs is the main cause of the high levels existent in plasma of cancer patients. Bryant and colleagues [113] have described the differential expressions of several EVs-related miRNA in serum and plasma (Further reviewed in [113]). Due to their strong role in cancer pathogenesis and its biological compatibilities, such as its ability to cross the blood-brain barrier, exosomes are considered to be viable candidates for a range of therapeutic and clinical applications.

## 3. Future Challenges and Conclusions

Early detection of cancer is pivotal in order to improve overall survival rates. Presently, there is an increasing tendency of studies based on the clinical value of detecting CTCs, ctNAs, and exosomes in peripheral blood, plasma, and serum from cancer patients. Currently, tumor detection is clinically confirmed using the traditional biopsy, which has detrimental effects on the patient. Thus, minimally invasive methods would be remarkably advantageous to the diagnosis and prognosis of cancer, and the subsequent development of patient-specific targeted treatments.

The optimal sensitivity and specificity will include a combination of CTC, ctDNA, miRNA, and exosomes detection and analysis from a patient’s blood specimen.

Recent technological advances have discovered that by combining the currently used molecular profiling with liquid biopsy-based approaches, it would lead to many integrated systems for biomarker capture, detection, and analysis using the same assay. In order to broaden the understanding of the cancer metastasis and its molecular landscape, a real-time tracking of the development of the tumor dynamics is required; for this reason, liquid biopsy-based approaches provide a valuable tool for minimally invasive diagnosis and monitoring [11]. However, there are still some concerns that need to be answered: Are the ctNAs sequences a clear clue about the stage of the cancer? Is the information given by the sequences present in the ctNAs, the same as the ones inside the exosomes and CTCs? Considering that ctNAs come from dead cells in contrast to the DNA existing within the exosomes, which will try to colonize new body regions, or the one inside CTCs that is escaping from the primary tumor, what variability of information could be extracted from these different sources to help in the prognosis and patient treatment?

Exploiting liquid biopsy approaches in patient screening could provide a more comprehensive view of tumor heterogeneity, including aggressiveness and the overall molecular landscape.

In conclusion, the next generation of liquid biopsy studies will be the key to definitively establishing the clinical applicability of patient-specific and blood-based molecular diagnostics. Liquid biopsy-based approaches will improve patient outcomes and OS; however, a key question remains: Will liquid-biopsy-based treatments represent a remarkable improvement in outcomes and, hence, patient’s health and economy?

## Figures and Tables

**Table 1:** Commercial techniques for NA purification.

Method	Principle	Comment	Reference
QIAamp DNA Mini Kit	NA purification based on a silica membrane	Rapid purification of high-quality DNA	[51]
		Consistent, high yields	
	DNA isolation, including of genomic, mitochondrial, viral, among others.	Contaminants and inhibitors removal	
QIAamp DSP Virus Spin Kit	Copurification of NA, based on a silica membrane, from human plasma serum.	Rapid universal viral NA purification	[52]
		High-quality viral NAs	
		Elution volume: 20–150 µL	
		Minimal risk of cross contamination	
NucleoSpin^®^ Plasma XS	Rapid purification of ctDNA from human plasma and serum, based on a silica membrane.	High recovery (DNA > 50 bp)	[53]
		Elution volume: 5 μL	
		Concentrated DNA, even if diluted	
		Ready-to-use DNA for downstream	
Agencourt Genfind v2	Isolation and purification of DNA from whole blood and serum.	Faster separation, easier manipulation and simple automation.	[54]
	Paramagnetic bead isolation for high recovery of DNA.	The method can be run manually in a 2 mL tube format	
